# Early evaluation predicts pain relief of irradiated bone metastases: a single-center prospective study

**DOI:** 10.1186/1472-684X-12-12

**Published:** 2013-03-13

**Authors:** Pierre Truntzer, David Atlani, Marius Pop, Jean-Baptiste Clavier, Sébastien Guihard, Catherine Schumacher, Georges Noel

**Affiliations:** 1 Radiation department, against cancer center Paul Strauss, 3, rue de la porte de l’hôpital BP42, Strasbourg cedex, 67065, France; 2 Radiation department Pasteur Hospital, Colmar, 68000, France

**Keywords:** Radiation therapy, Bone metastasis, Efficacy, Pain, Evaluation

## Abstract

**Background:**

Radiation therapy is a well-recognized, effective modality used for palliative care. Most studies completed to date have endpoints of one month or greater after treatment completion. This study analyzed the response rates at different time points during the first month after treatment.

**Methods:**

From May 2010 to November 2011, 61 patients treated for 74 metastases were included in the study. The end points were defined as the completion of treatment (CT) and d8, d15 and d30 after the completion of treatment. The response rate was measured by the worst pain in the last 24 hours and the administered opioid dose. Patient assessment was performed during consultations and phone appointments.

**Results:**

The overall response rate significantly improved from the CT (38%) to d8 (53.8%), d15 (53.8%) and d30 (57.1%) (respectively p < 0.001; p < 0.001 and p = 0.001). The improvement peaked at d8. Patients responding to the treatment at d8 had a significative longer pain relapse free survival (PRFS) compared to patients not responding (3.38 weeks vs 0.3 weeks; p < 0.001). From the beginning of treatment to the CT and at d8 , d15 and d30, oral morphine equivalent dose (OMED) did not significantly differ. However, the pain decrease did not result in a performance status improvement, which declined over time (p < 0.001).

**Conclusion:**

Radiation therapy is an efficient treatment method for providing pain relief. This relief peaked at d8 after treatment, and the response at d8 is predictive of the response at 4 weeks. Pain management alone is not enough to improve performance status; further studies are needed to evaluate a more global supportive care approach.

## Background

Approximately 50% of patients with cancer present metastases at the initial presentation or at relapse. Bone is one of the most frequent metastasis locations, particularly for lung, breast, and prostate cancers. Pain is the most common and debilitating symptom of bone metastases. Pain alters patient quality of life and requires specific support. This support includes motor function improvement and local or systemic treatment. Treatment may consist of the concomitant or alternate administration of analgesics, chemotherapy, hormone therapy, bisphosphonates, surgery, interventional radiology, and radiotherapy.

External beam irradiation for painful bone metastases has been well established by many prospective trials and can lead to pain relief and/or reduce analgesic consumption. Several radiation treatment schedules with similar effectiveness have been described. The most used schedules deliver 30 Gy in 10 fractions of 3 Gy, 20 Gy in 5 fractions of 4 Gy or a single fraction of 8 Gy [1-7]. An updated meta-analysis did not find any differences in the overall response rates between multi- and single fractionation (61% and 60%, respectively) [8]. Stereotactic beam radiation therapy (SBRT) and intensity-modulated radiotherapy (IMRT) have proved their effectiveness in spinal bone metastasis irradiation and re-irradiation [9-12]. A recent review showed that local control was achieved in 87% of cases [13].

The response to irradiation has been evaluated using many different types of measure. Visual analogue scales, a numeric scale, a Brief Pain Inventory, the dose of analgesics and the requirement for re-irradiation are the most frequently used [14]. Furthermore, the follow-up evaluation period after the completion of radiotherapy is variable in all trials and retrospective studies. Currently, evaluations at one or two months after irradiation completion are used to predict the definitive response to radiotherapy. However, this waiting time can be considered as too long to efficiently manage painful metastasis using re-irradiation, particularly as the life expectancy of these patients is short.

In this study, we report the response rate at the end of treatment and one, two and four weeks after treatment to determine whether an earlier evaluation could predict a definitive response.

## Methods

From May 2010 to October 2011, patients referred to the radiation department of the Regional Cancer institute Paul Strauss for painful bone metastases were eligible to participate in this study examining the response after irradiation. The inclusion criteria were age above 18, ability to understand and speak French and consent to participate to the study. This study was approved by our institutional review board (committee against pain CLUD). Patients who underwent previous surgery, cementoplasty, or in-field irradiation and remained in pain were also included. Clinical evaluations were performed at the first radiotherapy consultation (beginning of treatment, BT) and at the completion of treatment (CT). Patients provided informed consent. A visual analogue scale (VAS) strip was given to each patient to evaluate their pain at home after being instructed on how to use it. On days 8, 15 and 30 after the CT, evaluations were performed during a scheduled phone appointment. The interviewer (PT) used a home survey to obtain the patient information.

The information collected at the first consultation included age, gender, primary cancer location, pathology, painful bone metastasis location, performance status, steroid treatment, analgesic morphine taken in the last 24 hours converted to the oral morphine equivalent dose (OMED) and local treatments received previously [15]. On d8, d15 and d30, OMED, performance status, and the minimum, mean and maximal pain evaluations in the past 24 hours (assessed using the VAS) were reported. The adverse effects of radiation therapy were reported according to the CTCAE v4. Patients who did not answer the phone were considered missing but were not excluded from the study.

The radiotherapy response rates were evaluated using the international bone metastases consensus group (IBMCG) criteria (Table 1).

**Table 1:** The response rate to radiotherapy according to the IBMCG criteria

**Responders**	
**CR**	Pain reduction by two scores or more to zero **and** OMED stable or reduced
**PR**	Pain reduction by two scores or more **and** OMED stable or reduced
	Stable pain a**nd** OMED reduction by 25% or more
**Non-responders**	
**PD**	Pain increase by two scores or more **and** OMED stable or increased
	No change in pain **and** OMED increased by 25% or more (or start of morphine use after baseline)
**SD**	Stable pain **and** stable OMED
**UR**	Other cases

*Abbreviations*: CR: complete response; PR: partial response; PD: progressive disease; SD: stable disease; UR: undetermined response.

The treatment was performed using linear accelerators with 6 MV or 15 MV photon beams. The TomoTherapy Hi-ART and Novalis TX systems were used for IMRT and SBRT treatment planning. The time period between the first consultation and the first irradiation session did not exceed 2 weeks.

### Statistical analysis

The response rates at the different end points were compared for each patient: i) between the BT and CT, BT- d8, BT-d15 and BT-d30, ii) between the CT and d8, CT- d15 and CT-d30 iii) between d8- d15 and d8-d30, iv) between d15 and d30. Only patients with an available response rate at the compared end points were analyzed, and if the information was missing, the patient was excluded from the statistical analysis at the corresponding end point. Responders were patients with a complete response (CR) or a partial response (PR); patients with stable disease (SD), progressive disease (PD) and undetermined responses (UR) were considered as non-responders. The distributions of the Karnofsky performance status (KPS) (< 70% and ≥70%) and World Health Organisation performance status (WHO PS) (<2 and ≥ 2) at each evaluation were analyzed by the χ^2^ test. The nonparametric Wilcoxon test was used to compare repeated measures of pain score and OMED. A pain relapse free survival (PRFS) was calculated for the patients responding at every end points from the CT to d30 (range from 0 to 4 weeks). PRFS was analysed using a Kaplan Meyer method. *p* <0.05 was considered significant. Data were analyzed using SPSS version 20 (IBM Software).

## Results

Between May 2010 and November 2011, 61 patients were enrolled in the study. The general characteristics of the patients are summarized in Table 2. These 61 patients were treated for 74 metastatic locations: 31 were spinal (42%), 21 were pelvic (28%), 10 were long bones (13%) 8 were scapula (11%), 3 were rib, and 1 was mandibular. Forty-nine patients were treated for one metastatic location (80%), 11 for two (18%) and 1 patient for three.

**Table 2:** General patient characteristics before irradiation

	**N**	**%/ value**		**N**	**%/ value**
**Gender**			**Median KPS (min-max) at BT**		70 (30–100)
Male	37	60.7	**Median WHO-PS(min-max) at BT**		2 (0–4)
Female	24	39.3	**Treatment before RT**		
**Median Age (min-max)**		65 (43–88)	Surgery	10	13.6
**Primary cancer site**			Cementoplasty	9	12.2
Lung and pleura	20	32.8	RT	5	6.8
Breast	12	19.7	RT/Surgery	1	1.4
Kidney, prostate	10	16.4	No treatment	49	66.1
Gastrointestinal	7	11.4	**Radiation treatment modality**		
Head & Neck	3	4.8	30 Gy; 10 × 3 Gy	41	55.4
Unknown	2	3.2	20 Gy; 5 × 4 Gy	14	18.9
Other	7	11.4	8 Gy; 1 × 8 Gy	6	8.1
**Metastases localization**			23.31 Gy; 3 × 7.77 Gy	7	9.5
Spinal	31	41.9	Other	6	8.1
Pelvis	21	28.4	**Number of metastases**		
Long bone	10	13.5	1	49	80
Scapula	8	10.8	2	11	18
Other	4	5.4	3	1	2

*Abbreviations*: KPS: karnofsky performance status; RT: radiotherapy; WHO PS: world health organization performance status.

Forty-nine metastatic sites were not treated previously (66%). Twenty five metastases were treated before the present irradiation, within a minimum of one month. Ten metastases (14%) were operated on (decompressive, stabilization), 9 (12%) received cementoplasty, 5 (7%) were irradiated, and 1 patient underwent surgery and radiotherapy. The six patients who previously received irradiation presented metastases located in spine for four and in pelvis for two metastases. The irradiation schedules before re-irradiation were 8 Gy in a single fraction (one patient), 24 Gy in 6 fractions of 4 Gy (one patient), 30 Gy in 10 fractions of 3 Gy (two patients), and 36 Gy in 12 fractions of 3 Gy (two patients).

Concerning the current irradiation, 66 bone metastases (89%) were treated with 3D conformational radiotherapy. Seven spinal metastases (9.5%) were treated with SBRT (re-irradiation in 4 cases). One metastasis was treated with IMRT. For 62 treatments (91%), the dose schedules were 8 Gy in a single fraction, 20 Gy in 5 fractions of 4 Gy and 30 Gy in 10 fractions of 3 Gy. The SBRT dose was 23.31 Gy in 3 fractions of 7.77 Gy delivered on days 1, 3 and 5. Other fractionations were proposed in 5 patients.

All patients included completed the treatment, and an evaluation at the completion of treatment was performed in 50 patients (82%). On days 8, 15 and 30, evaluations were obtained from 39 (64%), 39 (64%), and 42 (69%) patients, respectively. The reasons for no reply included unreachable by phone, death or refusal to continue the study (Table 3). Patients not responding at d30 had a statistically significant worse WHO-PS (3 vs 2; *p =* 0.018), but age, gender, VAS and OMED weren’t statistically different. The response rate to the treatment, median worst pain, median WHO-PS, KPS and OMED are presented in Tables 4 and 5.

**Table 3:** The reasons for non-evaluation

	**CT**	**D8**	**D15**	**D30**
**Unreachable by phone**	11 (12.1%)	20 (32.8%)	20 (32.8%)	13 (21.7%)
**Dead**	0	1	1	5 (8.3%)
**Refusal to continue the study**	0	1	1	1
**# of patients**	**11 (18%)**	**22 (36.1%)**	**22 (36.1%)**	**19 (31.1%)**

*Abbreviations*: CT: completion of treatment.

**Table 4:** Response rates

	**CT**	**D8**	**D15**	**D30**
**Responders**	19 (38%)	21 (53.8%)	21 (53.8%)	24 (57.1%)
** CR**	4 (8%)	4 (10.3%)	6 (15.3%)	7 (16.7%)
** PR**	15 (30%)	17 (43.5%)	15 (38.5%)	17 (40.4%)
**Non-responders**	31 (62%)	18 (46.2%)	18 (46.2%)	18 (42.9%)
** SD**	20 (40%)	12 (30.8%)	10 (25.8%)	7(16.7%)
** PD**	7 (14%)	4 (10.3%)	6 (15.3%)	7 (16.7%)
** UR**	4 (8%)	2 (5.1%)	2 (5.1%)	4 (9.5%)
**Total patients**	**50**	**39**	**39**	**42**

*Abbreviations*: CR: complete response; PR: partial response; PD: progressive disease; SD: stable disease; UR: undetermined response.

**Table 5:** The intensity of the worst pain, OMED, performance status, and Karnofsky index at baseline, the CT, d8, d15 and d30 after radiotherapy treatment

	**BT**	**Evaluation time**	**Results**	P*
**Median worst pain (range)**	7 (1–10)	CT	5 (0–10)	0.001
		D8	3 (0–10)	<0.001
		D15	3 (0–10)	<0.001
		D30	3 (0–10)	<0.001
**Median/Mean OMED (mg) (range)**	40/90 (0–920)	CT	40/111.1 (0–720)	0.93
		D8	30/65.7 (0–380)	0.64
		D15	30/75.9 (0–480)	0.47
		D30	40/91.2 (0–570)	0.96
**WHO PS (range)**	2 (0–4)	CT	2 (0–4)	0.003
		D8	2 (0–4)	0.004
		D15	2 (0–4)	0.011
		D30	2 (0–4)	0.003
**KPS (range)**	70 (30–100)	CT	70 (30–100)	0.001
		D8	70 (30–90)	0.001
		D15	70 (30–100)	0.006
		D30	70 (30–100)	0.002

*Abbreviations*: BT: beginning of treatment; CT: completion of treatment; KPS: karnofsky performance status; OMED: oral morphine equivalent dose; WHO PS: world health organization performance status.

### Analysis of visual scale

Using the visual scale, the patients had a significant decrease in the median maximal pain from the BT to the CT from VAS 7 to VAS 5, respectively (*p* = 0.001). From the BT to d8, d15 and d30, the VAS decreased to 3 and remained statistically significant (*p <* 0.001 at each evaluation). There was no significant difference in the maximal VAS from the CT to d8, d15 or d30 (*p* = 0.115; *p* = 0.088; *p* = 0.072, respectively), from d8 to d15 or d30 (*p* = 0.441 and *p* = 0.393, respectively) or from d15 to d30 (*p* = 0.345).

### Analysis of OMED

The OMED did not significantly differ between each interval of evaluation. The mean OMEDs at the BT, the CT, d8, d15 and d30 were 40 mg, 40 mg, 30 mg, 30 mg and 40 mg, respectively (*p =* 0.93 between BT and CT, *p* = 0.64, for CT-d8, *p* = 0.47 for CT-d15, *p* = 0.96 for CT-d30 and *p* = 0.96 for BT-d30).

### Analysis of IBMCG

The overall response rates at CT, d8, d15 and d30 were 38%; 53.8%; 53.8% and 57.1% respectively. The CR rates at CT, d8, d15 and d30 were 8%, 10.3%, 15.3% and 16.7% respectively. Using the IBMCG criteria, there were significant improvements (CR, PR) from the CT to d8, d15 and d30 (*p* < 0.001; *p* < 0.001 and *p* = 0.001, respectively), from d8 to d15 and d30 (*p* < 0.001 and *p* = 0.001, respectively), and from d15 to d30 (*p* = 0.002). Univariate analysis using Kaplan Meyer method highlighted PRFS for patients having a response at d8 was significantly improved compared to patients not responding: respectively 3,38 weeks vs 0,3 weeks (p < 0,001). This result suggests response at d8 is predictive of response at d30.

### Performance status (WHO PS and KPS)

Compared to the BT, WHO PS and KPS were significantly decreased at the CT, d8 and d30 (*p* < 0.001). However, at d15, the data showed a transitory improvement in WHO-PS and KPS (*p <* 0.001).

### Irradiation side effects

The side effects of radiotherapy included dermatitis, nausea, vomiting, diarrhea and dysphagia in 11 patients (18%) at the CT, 9 (15%) patients at d8, 9 (15%) patients at d15 and 9 (15%) patients at d30 (Table 6). According to CTCAE v4, only one patient developed grade 3 toxicity at the CT and d8; the remaining patients experienced grade 2 or less toxicities. After d8, the toxicities were grade 2 or less.

**Table 6:** Radiation therapy side effects at different end points

	**CT (# patients)**	**D8 (# patients)**	**D15 (# patients)**	**D30 (# patients)**
Nausea,vomiting	3	2	1	1
Grade 1	2	1	1	1
2	0	1	0	0
3	1	0	0	0
Diarrhea	2	1	2	2
Grade 1	1	0	2	2
2	1	1	0	0
3	0	0	0	0
Dysphagia	5	5	5	5
Grade 1	3	1	4	4
2	2	3	1	1
3	0	1	0	0
Dermatitis	1	1	1	1
Grade 1	1	1	1	1
2	0	0	0	0
3	0	0	0	0
Total	11	9	9	9

*Abbreviations* CT: completion of treatment.

## Discussion

Several consensus task forces have attempted to recommend tools or methods for following and evaluating patients with bone metastases [16,17]. One of the recommendations was to assess pain at 2 months after the CT [18]. However, we believe that this amount of time is too long for patients in pain who may have a short life expectancy. Our study clearly showed that an evaluation at d8 is sufficient for proposing re-irradiation or another treatment for remaining painful metastases, as patients not responding at this end point have significantly lower PRFS. Another proposal was to perform re-irradiation at least one month after the previous irradiation. However, with new techniques, such as IMRT or SBRT, which can shield organs at risk (particularly the spinal cord), a higher efficient dose can be delivered, dramatically increasing the chance of controlling pain with a very low risk of complications.

This study showed phone evaluation’s feasibility to assess response to an antalgic irradiation, in order to be able to propose an adequate care of the pain. This method allows the assessment of patients at home, which is more comfortable and avoids travel. Phone evaluations can be performed by a radiation oncologist or a trained nurse. In 2004, a study assessing 830 patients by phone retrieved a higher percent of patients reachable by phone at 4 weeks (57%) after treatment completion with a steady decrease through 12 weeks (48%) [19]. Our results are comparable to that study, with 72% of patients reachable at the CT and 64-69% at d8, d15 and d30. As in a previously published series, the main cause of no reply was death or hospitalization [19].

In 2004, Chow and *al*. analyzed 580 patients’ response rates to radiation therapy using pain intensity scale and analgesic consumption [20]. In this study the overall response rates at 1, 2 and 4 weeks were 41%, 41% and 39% respectively. The CR rates at 1, 2 and 4 weeks were 32%, 28% and 26% respectively. In our study the overall response rates at 1,2 and 4 weeks were 38%, 53.8%, 53.8% and 57.1% respectively and CR at 1,2 and 4 weeks were 8%, 10.3%, 15.3%, and 16.7%. Our results are comparable to previously published results using the same evaluation method (IBMCG) [20] and time at evaluation (one month) [21].

Despite the improvement of the overall response to pain after radiation therapy, our study showed a worsening of the performance status. Patients had an altered performance status with a median WHO PS of 2 (0–4) at the BT. In several previous studies and trials, most patients had a WHO PS ≥ 2 [4,5]. Unfortunately, patients with a very poor performance status at the BT, have a low probability of improving their *performance status* because many factors contribute to this status. However, one study showed that such patients with a short life expectancy may benefit from analgesic irradiation [22].

This study has some limitations: the population is mixed concerning metastatic localization, performance status, radiation therapy schedule, and radiation technique (RTC 3D or SBRT).

## Conclusions

The prospective study demonstrated that radiation therapy is a quick and efficient treatment of bone metastasis pain. A phone evaluation of pain relief is feasible in practice. The evaluation of pain at d8 after the CT is predictive of pain relief thereafter. Thus, if needed, treatment decisions can be made earlier in the patient’s disease course. However, a good pain response is not strictly correlated to performance status.

## Abbreviations

VAS: Visual analogue scale; BT: Beginning of treatment; CR: Complete response; CT: Completion of treatment; KPS: Karnofsky performance status; IBMCG: International bone metastases consensus group; IMRT: Intensity-Modulated radiation therapy; OMED: Oral morphine equivalent dose; PD: Progression disease; PR: Partial response; PRFS: Pain relapse free survival; RT: Radiation treatment; SD: Stable disease; SBRT: Stereotactic beam radiation therapy; UR: Undetermined response; WHO PS: World health organisation performance status.

## Competing interests

The authors declare no conflicts of interest.

## Authors’ contribution

PT included patients, interviewed patients by phone, conducted statistical analysis, and drafted the manuscript. GN included patients, conceived of the study and participated in its design and coordination and helped to draft the manuscript. DA, MP, JBC, SG, CS included patients. All authors read and approved the final manuscript.

## Pre-publication history

The pre-publication history for this paper can be accessed here:

http://www.biomedcentral.com/1472-684X/12/12/prepub
